# Co-occurrence of Congenital Osteogenesis Imperfecta and Maternal Antiphospholipid Syndrome: A Novel Case Report

**DOI:** 10.7759/cureus.88164

**Published:** 2025-07-17

**Authors:** Mouna Zouine, Salma Sekkat, Houda Moustaide, Saad Benkirane, Abdallah Oulmaati

**Affiliations:** 1 Pediatrics and Neonatology Department, Mohammed VI University Hospital, Faculty of Medicine and Pharmacy, Abdelmalek Essaâdi University, Tangier, MAR; 2 Medical Engineering Department, Mohammed VI University Hospital, Faculty of Medicine and Pharmacy, Abdelmalek Essaâdi University, Tangier, MAR; 3 Obstetrics and Gynecology Department, Mohammed VI University Hospital, Faculty of Medicine and Pharmacy, Abdelmalek Essaadi University, Tangier, MAR

**Keywords:** antiphospholipid syndrome, fetal hereditary disorder, inherited disorders, osteogenesis imperfecta, zoledronic acid

## Abstract

Osteogenesis imperfecta (OI), the second leading cause of congenital osteopenia, is a hereditary connective tissue disorder with a rare occurrence. It results from *de novo* or inherited mutations, justifying the search for a family history, particularly cases of consanguinity or similar conditions. However, its association with maternal pathologies, especially those of autoimmune origin, remains unexplored in the literature, based on our research. Antiphospholipid syndrome (APS) primarily leads to major obstetric complications: miscarriages, thromboses, and fetal growth restrictions. But is there an established link between maternal APS and fetal OI? We present here a case of a newborn with OI, whose mother's pregnancy was complicated by maternal APS, illustrating a potential association that warrants further investigation.

## Introduction

Osteogenesis imperfecta (OI) is a group of inherited disorders characterized by excessive bone fragility, often leading to multiple fractures with minimal or no trauma. It is primarily caused by mutations in the COL1A1 or COL1A2 genes, which encode the pro-α1 and pro-α2 chains of type I collagen. These chains assemble into a triple helix, forming type I collagen, the primary structural protein in bones and connective tissues. This collagen is also found in other tissues, including the skin, tendons, ocular sclera, and dentin [1].

The severity and progression of OI vary widely among individuals, but the most significant consequence remains reduced bone strength, resulting in a high fracture risk and complications stemming from abnormal collagen production [1,2]. To date, hundreds of distinct mutations in these genes have been linked to different OI phenotypes. Most cases arise from de novo mutations, though parental germline mosaicism can occasionally be the cause.

These mutations may be triggered by various factors that interfere with fetal DNA. Could maternal autoimmune disease be one such factor?

We present a case of a newborn with congenital OI born to a mother diagnosed with antiphospholipid syndrome during pregnancy.

## Case presentation

We present a case of a male newborn, born to a non-consanguineous couple with no family history of connective tissue disorders or similar cases. The pregnancy was monitored and carried to 39 weeks + four days of gestation (last menstrual period). Maternal history was G5P3 (five pregnancies: three live births and two first-trimester miscarriages at eight weeks of gestation, with no etiological workup). Current pregnancy complications: at 24 weeks of gestation, the mother developed pulmonary embolism, requiring anticoagulant therapy. Diagnosis of antiphospholipid syndrome was established based on clinical criteria with the following immunological findings (Table 1).

**Table 1:** Immunological workup results of the mother at 24 weeks of gestation. In this patient presenting with pulmonary embolism at 24 weeks of gestation (WG) and a history of miscarriages, the lupus anticoagulant (LA) assay revealed an LA1 ratio (M/T) of 1.63, confirming the presence of a lupus anticoagulant. This result, in an obstetrical and thrombotic context, strongly suggests obstetric-vascular antiphospholipid syndrome (APS). The triad of thrombosis, fetal loss, and LA positivity is highly indicative of APS.

Requested antibodies	Results	Reference ranges			Interpretation
		Negative	Indeterminate	Positive	
Anti-cardiolipin antibodies	IgG < 3 U/mL	<7 GPL-U/mL	7–9 GPL-U/mL	>9 GPL-U/mL	Negative
	IgM < 3 U/mL	<7 GPL-U/mL	7–9 GPL-U/mL	>9 GPL-U/mL	Negative
Lupus anticoagulant	LA1 control (T): 68.60 sec	-	-	-	-
	LA2 patient (M): 45.50 sec	-	-	-	-
	LA1 ratio (M/T): 1.63	-	<1.20	-	Presence of an inhibitory effect
Anti-β2-glycoprotein I antibodies	IgG: 4 U/mL	<12 U/mL	12–18 U/mL	>19 U/mL	Negative
	IgM < 3 IU/mL	<12 U/mL	12–18 U/mL	>19 U/mL	Negative

**Figure 1:**
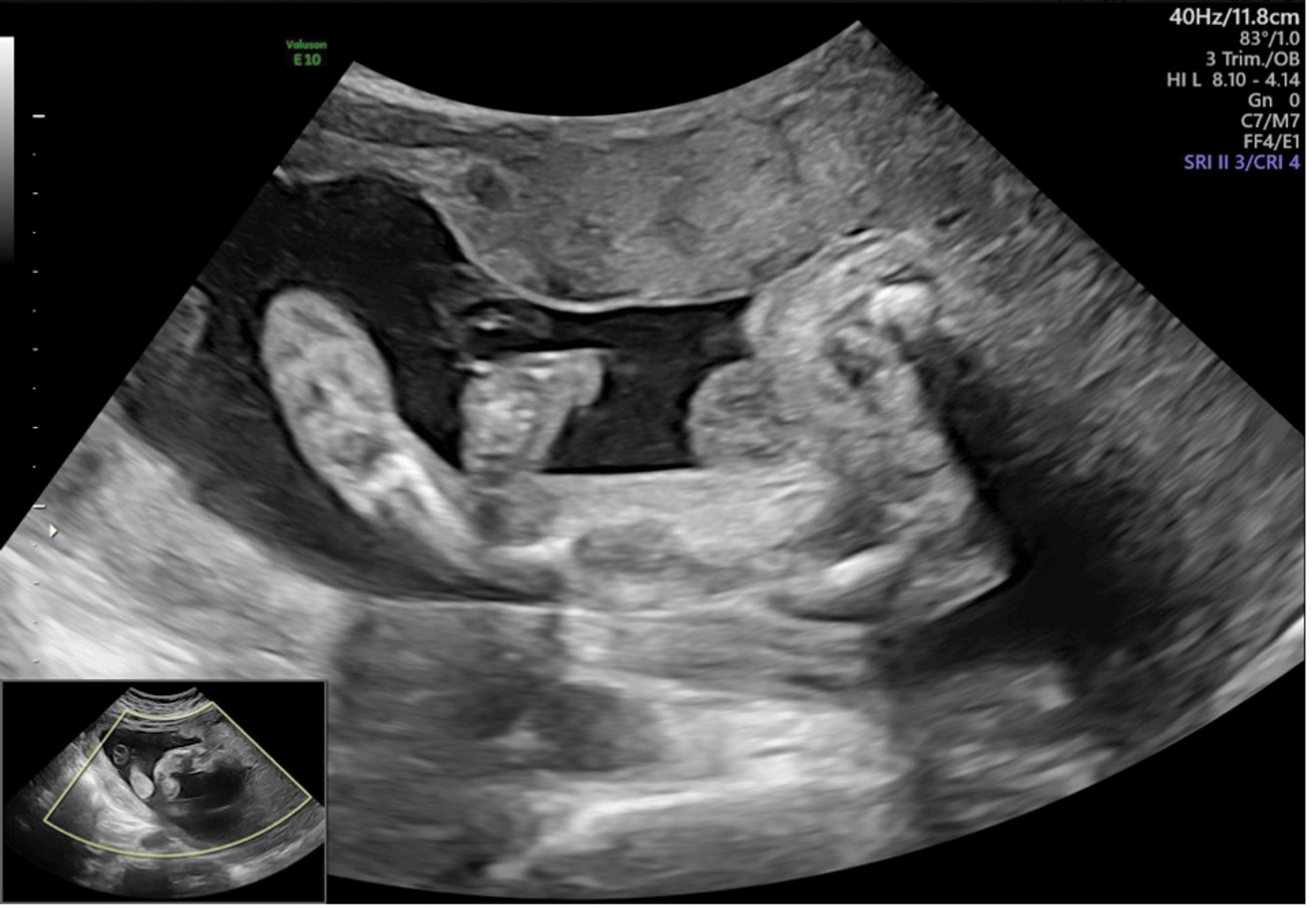
Antenatal ultrasound. Antenatal ultrasound demonstrates multiple structural anomalies, notably involving the femurs and feet, including clubfoot deformities.

The second-trimester obstetric ultrasound showed a malformation of both lower limbs consistent with clubfoot, with femoral curvature, and the third-trimester ultrasound revealed moderate hydrocephalus (Figure 1).

**Figure 2:**
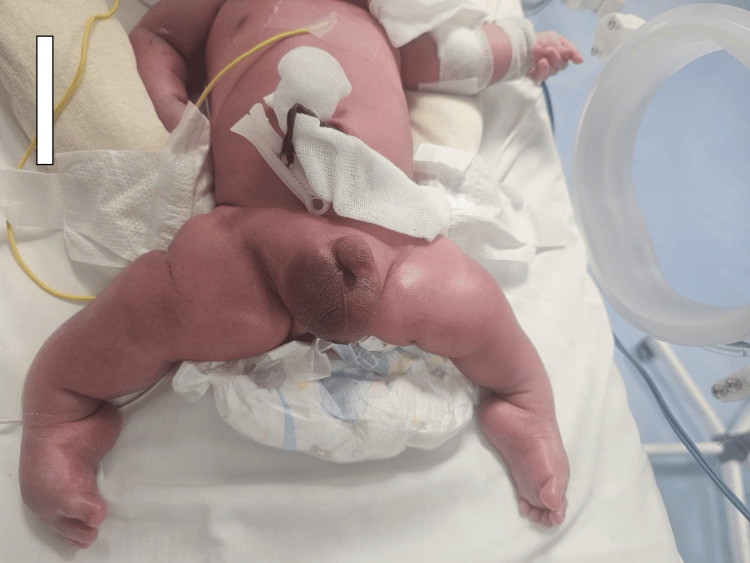
Full-body frontal morphological photograph, highlighting the architectural deformities of the upper and lower limbs. Three-dimensional deformities of the upper and lower limbs. This complementary visual analysis enables clinico-radiological correlation of morphostatic alterations.

Delivery occurred vaginally with a triple nuchal cord and meconium-stained amniotic fluid. Apgar scores were 3/10 at one minute and 8/10 at five minutes. The newborn's examination revealed a hypotonic infant with tachypnea and respiratory distress, scored at 4/10 according to the Silverman-Anderson score, with initial oxygen saturation at 75% on room air, improving to 98% under continuous positive airway pressure (CPAP). The upper and lower limbs were short and deformed, with bilateral clubfoot (Figure 2).

**Figure 3:**
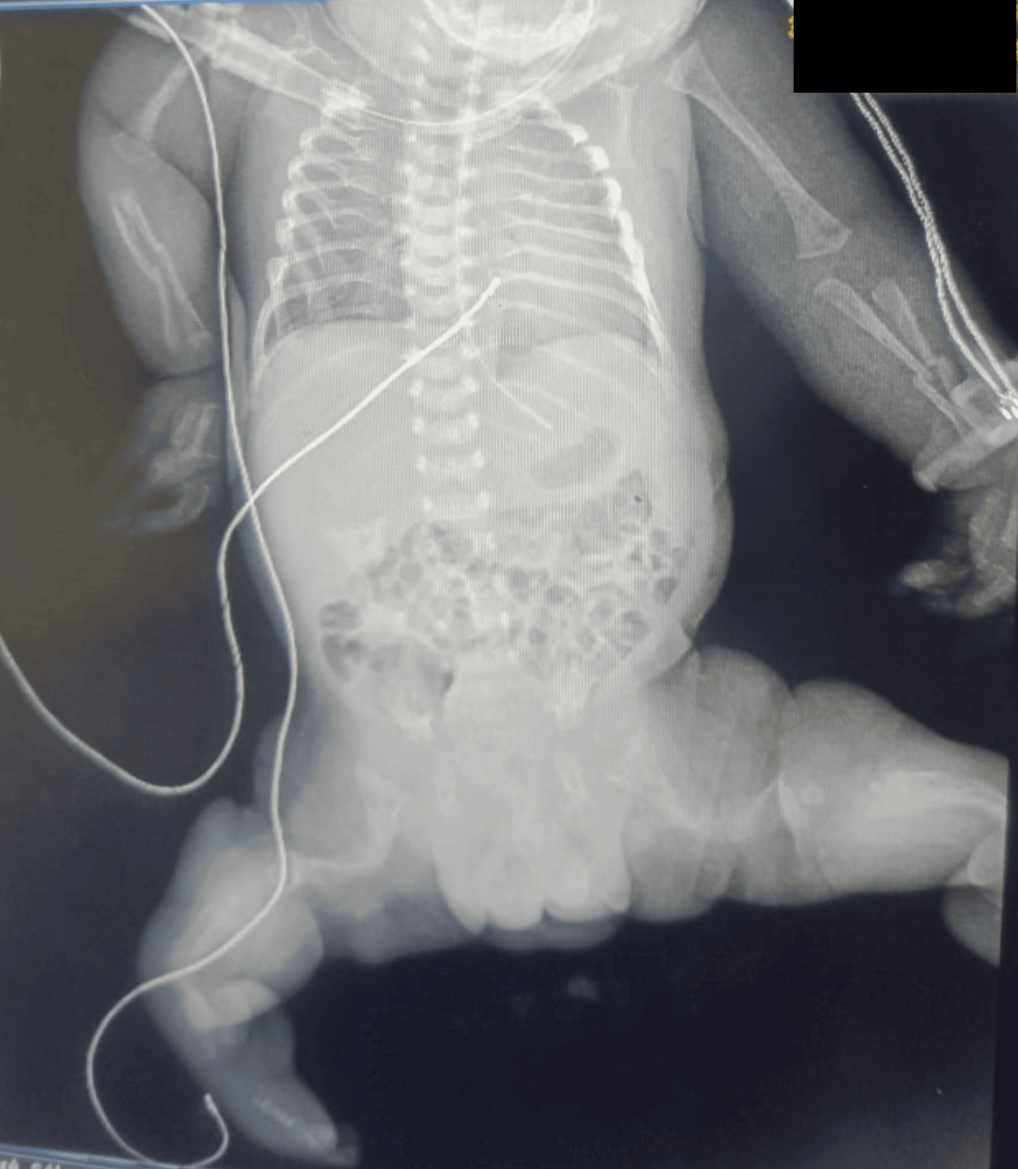
Full-body frontal radiography in supine position. Multiple bilateral fractures of the radius and ulna, accompanied by severe bone deformities and pronounced bowing, predominantly affecting the femurs and tibias. Of particular note is the presence of a malunited fracture in the right tibia.

Macrocephaly was also noted, with a head circumference of 39 cm. Limb and chest radiographs demonstrated bone deformities with multiple fractures of varying ages in the upper and lower limbs as well as the ribs (Figure 3).

The neonatal and maternal calcium-phosphorus metabolic panels were both normal. Genetic testing identified a heterozygous pathogenic variant in the COL1A2 gene, consistent with osteogenesis imperfecta.

The therapeutic management, combining antibiotic therapy and CPAP ventilation, stabilized the newborn's respiratory status. The administration of zoledronic acid at a dosage of 0.025 mg/kg, initiated before obtaining the genetic results, promoted accelerated bone consolidation. No calcium supplementation was administered during zoledronic acid treatment, as the pre-therapeutic calcemia was around 2.2 mmol/L. Oral calcium supplementation (0.25 mmol/kg three times daily) was initiated immediately after zoledronic acid administration. However, 48 hours after the infusion, generalized tonic-clonic seizures occurred in the context of severe hypocalcemia, despite calcium supplementation. The seizures were controlled with anticonvulsant therapy and correction of hypocalcemia, leading to normalization of the clinical picture.

Follow-up and outpatient monitoring revealed a new isolated humeral fracture. Despite axial and peripheral hypotonia observed on examination, the nine-month evaluation showed no delays in socio-cognitive abilities: normal visual fixation and tracking, and preserved interactive responses (social smiling, babbling), suggesting a purely motor etiology. Accurate weight assessment was challenging due to the presence of immobilizing casts, which limited measurement precision. Regarding height progression, recorded data indicated a birth length of 48 cm (< -3 SD), followed by steady growth: 56 cm at three months (-3 to -2 SD), 62 cm at six months (-3 to -2 SD), and 67 cm at nine months (-2 SD). Although this trend demonstrated consistent growth, the values remained below -2 SD by nine months of age, a finding that may be partially attributable to tibial bowing.

## Discussion

OI is a highly complex disease characterized by significant molecular heterogeneity. Several types can be distinguished based on the timing and/or severity of clinical presentation. The Sillence classification divides OI into four types. Type I: autosomal dominant inheritance; corresponds to a mild form with blue sclerae. Type II: autosomal dominant inheritance; represents the perinatal and lethal form. Type III: autosomal dominant inheritance; constitutes the severe form, with or without dentinogenesis imperfecta. Type IV: autosomal dominant inheritance; this form is clinically similar to type I but differs in its normal sclera coloration and greater severity.

The diagnosis primarily relies on clinical examination, making it essential to obtain a detailed family history of bone disorders. Laboratory tests are not highly informative for OI but are crucial to rule out other conditions, such as neonatal hyperparathyroidism and hypophosphatasia. A complete skeletal survey is useful to assess the number of fractures, including vertebral fractures, and the degree of limb deformities [3,4].

Although most genetic abnormalities result from inherited mutations, certain maternal conditions can indirectly affect the fetal genome, either through direct DNA damage or by disrupting fetal development. Autoimmune diseases, which disproportionately affect women, may complicate pregnancy, posing risks to both the mother and the fetus. Regardless of the specific autoimmune disorder, three key factors must be assessed during pregnancy: the immunological profile, disease activity, and visceral involvement. In the case we present here, the mother’s immunological profile was negative, yet the clinical picture, i.e., recurrent miscarriages and a pulmonary embolism episode, strongly suggests autoimmune pathology. Notably, neonatal lupus is the most significant fetal complication of maternal autoimmune diseases, particularly when SSA/SSB antibodies are positive [4]. APS is primarily responsible for placental insufficiency, characterized by disruptions in calcium-phosphate exchange and utero-placental vascularization. These pathophysiological alterations can lead to severe pregnancy complications, including spontaneous miscarriages, intrauterine fetal deaths, intrauterine growth restrictions, or preterm deliveries. To date, no causal relationship has been scientifically proven between maternal APS and the occurrence of fetal genetic mutations [5,6]. Current literature data indicate that maternal autoimmune disorders, including APS, do not appear to induce de novo mutations in the fetal genome. Despite the natural bidirectional transfer of genetic material and cells between maternal and fetal organisms during pregnancy, resulting in the postnatal persistence of fetal cells in maternal circulation (fetal microchimerism) and the presence of maternal cells in the newborn’s body (maternal microchimerism), current studies have not established a correlation between this microchimerism and the induction of fetal genetic mutations. Its primary role appears to be limited to modulating the neonatal immune response to maternal autoantigens without causing significant genomic alterations [7]. Moreover, in OI type III, as illustrated in this case report, intrauterine growth is severely restricted, and affected newborns present with numerous poorly healed fractures and pronounced deformities due to extreme bone fragility. Other clinical features include markedly shortened limbs and severe leg deformities, with hips flexed and thighs fixed in abduction and external rotation. The combination of a short trunk and a small thoracic cage predisposes infants to severe, progressive pulmonary insufficiency requiring ventilatory support, as seen in this newborn [8].

During this patient’s management, we faced significant medical and logistical challenges, including managing severe respiratory distress (which necessitated assisted ventilation immediately after birth), addressing both infectious complications and restrictive lung disease secondary to rib fractures, and implementing pain management with class II and III analgesics combined with immobilization of fractured limbs, a task that proved particularly challenging. Treatment with zoledronic acid (ZA) ultimately led to the reduction and eventual disappearance of chronic pain, increased muscle strength, and improved functional capacity [9]. ZA is a third-generation bisphosphonate whose superior potency allows for less frequent administration. It has been tested for several bone disorders, primarily in adults, but the efficacy and safety of ZA in very young children with OI have been less studied than those of pamidronate [10]. In 2004, Högler et al. [10] evaluated the use of ZA in 34 children following an initial dose of 0.02-0.025 mg/kg. This study reported a high incidence of flu-like symptoms (85%), hypocalcemia (74%), and hypophosphatemia (82%). Although a lower dose of 0.0125 mg/kg was associated with a reduced rate of hypocalcemia, comparable to that observed in children with OI treated with intravenous pamidronate, other studies reported lower rates of symptomatic hypocalcemia in children receiving ZA [11]. In 2022, Kumar et al. demonstrated that ZA, administered at 0.05 mg/kg every six months in children under five years old with OI, significantly reduced fractures, improved mobility, pain, and growth, with a favorable safety profile. After two years of treatment, the Clinical Severity Score (CSS) improved, confirming the efficacy and tolerability of ZA, making it a practical alternative to pamidronate due to its less frequent dosing regimen [12].

Although performed late, the genetic testing for OI in this newborn confirmed the diagnosis and guided the genetic counseling provided to the parents. Psychological support was also implemented due to their emotional distress.

## Conclusions

Osteogenesis imperfecta is a genetic disorder affecting the bone extracellular matrix. However, its association with maternal conditions such as lupus or antiphospholipid syndrome, as well as the impact of these autoimmune diseases on the bone genetics of their offspring, remains insufficiently explored.

## References

[1] Van Dijk FS, Sillence DO (2014). Osteogenesis imperfecta: clinical diagnosis, nomenclature and severity assessment. Am J Med Genet A.

[2] Shao YJ, Chen YM (2024). Parental autoimmunity and offspring risks of rheumatic diseases: a nationwide population-based study. Rheumatology (Oxford).

[3] Bishop N (2010). Characterising and treating osteogenesis imperfecta. Early Hum Dev.

[4] Andreoli L, Andersen J, Avcin T (2024). The outcomes of children born to mothers with autoimmune rheumatic diseases. Lancet Rheumatol.

[5] Costedoat-Chalumeau N, Guettrot-Imbert G, Leguern V (2012). Pregnancy and antiphospholipid syndrome. (Article in French). Rev Med Interne.

[6] Leveque L, Khosrotehrani K (2011). Can maternal microchimeric cells influence the fetal response toward self antigens?. Chimerism.

[7] Yimgang DP, Brizola E, Shapiro JR (2016). Health outcomes of neonates with osteogenesis imperfecta: a cross-sectional study. J Matern Fetal Neonatal Med.

[8] Hackley L, Merritt L (2008). Osteogenesis imperfecta in the neonate. Adv Neonatal Care.

[9] Senturk L, Gulec C, Sarac Sivrikoz T (2024). Association of antenatal evaluations with postmortem and genetic findings in the series of fetal osteogenesis imperfecta. Fetal Diagn Ther.

[10] Högler W, Yap F, Little D, Ambler G, McQuade M, Cowell CT (2004). Short-term safety assessment in the use of intravenous zoledronic acid in children. J Pediatr.

[11] George S, Weber DR, Kaplan P, Hummel K, Monk HM, Levine MA (2015). Short-term safety of zoledronic acid in young patients with bone disorders: an extensive institutional experience. J Clin Endocrinol Metab.

[12] Kumar C, Panigrahi I, Somasekhara Aradhya A, Meena BL, Khandelwal N (2016). Zoledronate for osteogenesis imperfecta: evaluation of safety profile in children. J Pediatr Endocrinol Metab.

